# Claustrum projections to prefrontal cortex in the capuchin monkey (*Cebus apella)*

**DOI:** 10.3389/fnsys.2014.00123

**Published:** 2014-07-03

**Authors:** David H. Reser, Karyn E. Richardson, Marina O. Montibeller, Sherry Zhao, Jonathan M. H. Chan, Juliana G. M. Soares, Tristan A. Chaplin, Ricardo Gattass, Marcello G. P. Rosa

**Affiliations:** ^1^Department of Physiology, Monash UniversityClayton, VIC, Australia; ^2^Department of Psychology and Psychiatric Medicine, Monash UniversityClayton, VIC, Australia; ^3^Programa de Neurobiologia, Instituto de Biofísica Carlos Chagas Filho, Universidade Federal do Rio de JaneiroRio de Janeiro, Brazil; ^4^Australian Research Council Centre of Excellence for Integrative Brain FunctionClayton, VIC, Australia

**Keywords:** claustrum, primate, cortical networks, neuroanatomy, prefrontal

## Abstract

We examined the pattern of retrograde tracer distribution in the claustrum following intracortical injections into the frontal pole (area 10), and in dorsal (area 9), and ventral lateral (area 12) regions of the rostral prefrontal cortex in the tufted capuchin monkey (*Cebus apella*). The resulting pattern of labeled cells was assessed in relation to the three-dimensional geometry of the claustrum, as well as recent reports of claustrum-prefrontal connections in other primates. Claustrum-prefrontal projections were extensive, and largely concentrated in the ventral half of the claustrum, especially in the rostral 2/3 of the nucleus. Our data are consistent with a topographic arrangement of claustrum-cortical connections in which prefrontal and association cortices receive connections largely from the rostral and medial claustrum. Comparative aspects of claustrum-prefrontal topography across primate species and the implications of claustrum connectivity for understanding of cortical functional networks are explored, and we hypothesize that the claustrum may play a role in controlling or switching between resting state and task-associated cortical networks.

## Introduction

Although it was first described over 200 years ago, the claustrum remains an enigma in modern neuroscience (Crick and Koch, 2005; Smythies et al., 2014). The convoluted geometry and difficult surgical approach to the claustrum, combined with its close proximity to the insula and putamen, have contributed to the uncertainty regarding claustrum function, as has the poor understanding of its cytoarchitectonic and chemoarchitectonic organization. The dearth of information regarding the claustrum is particularly acute among primate species. Although it has been shown that the claustrum has widespread reciprocal connectivity with the cerebral cortex, there is growing evidence for species differences in morphology, neurochemistry, and connectivity (reviewed in Baizer, 2014).

Claustrum connections to prefrontal areas have been examined in rodents (Vertes, 2004; Hoover and Vertes, 2007) and primates (Pearson et al., 1982; Tanne-Gariepy et al., 2002), as well as humans (Fernandez-Miranda et al., 2008; Milardi et al., 2013). In the rat, projections to the prelimbic area arise from the dorsal or insular portion of the claustrum, while projections to the infralimbic cortex are concentrated in the ventral (endopiriform) portion of the claustrum (Vertes, 2004). Hoover and Vertes (2007) expanded the range of observed rat prefrontal areas to include the anterior cingulate and frontal agranular areas, but reported a similar distribution of afferent projections to the earlier study, along with dense projections from the dorsal claustrum to anterior cingulate and frontal agranular areas.

In macaques, Pearson et al. (1982) and Tanne-Gariepy et al. (2002) examined claustrum afferents to lateral prefrontal areas, including areas 8, 9, 12, and 46, and to motor and premotor areas of frontal cortex. These studies showed that projections to area 46 were widespread, and extended along the majority of the rostral-caudal axis of the claustrum (this includes the injections of area 9 in Pearson et al., 1982). Projections to area 12 overlapped the distribution of area 9 connections, but extended more ventrally, especially in the more caudal portion of the claustrum. Afferent input from the claustrum to supplementary and premotor areas in both studies were segregated from prefrontal inputs along the dorsal-ventral axis of the claustrum, with less prominent separation of labeled cells along the rostral-caudal axis.

In humans, Fernandez-Miranda et al. (2008) described segregation of the claustro-cortical white matter tracts using a combination of cadaver dissection and diffusion tensor imaging, with clear separation of frontal and prefrontally projecting axons from those projecting to other cortical areas, e.g., temporal and parietal cortex. Despite advances in tractographic imaging methods (e.g., Milardi et al., 2013), non-human primates remain the best experimental model for detailed studies of connectivity of larger networks of cortical areas. It is therefore essential to understand the homology between identified cortical areas across species, in order to make accurate comparisons. To date, there have been few studies of claustro-cortical connections in New World monkey species.

The present study describes the claustral projections to the prefrontal cortex of the *Cebus* (capuchin) monkey, a species of New World monkey. The anatomy of *Cebus* monkey prefrontal cortex has recently been described in detail by Cruz-Rizzolo et al. (2011), including identification of cytoarchitectonic and myeloarchitectonic boundaries of cortical areas corresponding to those identified in macaques (Petrides and Pandya, 1999, 2002; Chaplin et al., 2013) and marmosets (another species of New World monkey; Burman et al., 2006; Burman and Rosa, 2009; Paxinos et al., 2012). We have previously reported that the dorsal and lateral portions of the frontal pole (area 10) of the marmoset receives a rich claustrum projection (Burman et al., 2011a,b).

## Materials and methods

Three adult *Cebus apella* monkeys were injected with fluorescent tracers, including fluororuby (FR, 10% in dH_2_0), fluoroemerald (FE, 10% in dH_2_0), diamidino yellow (DY, 2% in dH_2_0), and fast blue (FB, 2% in dH_2_0), at multiple locations in prefrontal and orbitofrontal cortex. Case details for each animal are summarized in Table 1. All surgical and experimental procedures were approved in advance by the Animal Ethics Committee of the Centro de Ciências da Saúde of the Universidade Federal do Rio de Janeiro (CEUA IBCCF189-06/16), and conformed to the guidelines of the Brazilian Federal Arouca law governing laboratory animal use and care, as well as the Australian Code of Practice for Care and Use of Animals for Scientific Purposes. Tracer injections and histological processing were conducted at the Instituto de Biofísica Carlos Chagas Filho, Rio de Janeiro, Brazil. Microscopic examination and data analysis were performed in the Department of Physiology of Monash University. Throughout this report, the numerical designations used for the various prefrontal areas conform to those of Cruz-Rizzolo et al. (2011). Stereotaxic location estimates are based on the Eidelberg and Saldias atlas (1960).

**Table 1:** **Case information and tracer injection locations**.

**Animal ID**	**Body weight (kg)**	**Sex**	**Hemisphere**	**Tracer**	**Amount (uL)**	**Location**
FR01	3.3	M	R	FB	0.4	Area 10
				DY	0.4	Area 10
				FE	1.0	Area 10
				FR	1.0	Area 10
FR02	3.0	M	R	FB	0.5	Area 10
				FE	1.0	Area 10
				DY	0.5	Area 12
FR04	3.0	M	R	FB	0.5	Area 9
				DY	0.5	Area 12o

*Summary of individual case data and injection targets. Recovery time for all animals was 2 weeks post-injection*.

### Tracer injections

All tracers were injected using a 1 μL Hamilton syringe. The animals were pre-medicated with atropine (0.15 mg/kg IM) and diazepam (0.5 mg/kg IM) and anesthetized with ketamine (30 mg/kg IM) and maintained using intramuscular ketamine and xylazine (1:5). All animals received peri-operative antibiotics (penicillin G, 300,000 IU, IM) and dexamethasone (0.3 mg/kg, IM).

A craniotomy was performed over the target regions of cortex, and the tracer was deposited in 50–100 nL increments over approximately 15 min. The micropipette tip was left in place for an additional 5–10 min following the last deposit, in order to minimize leakage of tracer into non-target areas. The injection into area 12o was accessed from the dorsal surface of the frontal cortex and intervening white matter. Tracer leakage along the needle track was minimized by slow withdrawal; however, it is possible that some contamination of the adjacent white matter occurred. After the final injection, the tip was withdrawn, and the bone flap excised during the craniotomy was replaced and cemented into place. The overlying tissue was sutured and the animal was allowed to recover until it could make spontaneous and coordinated movements, after which it was returned to the home cage. Each animal was carefully monitored during the 14 day post-injection survival period, during which analgesics and antibiotics were provided as required. At the end of the survival period, each animal was humanely euthanized with an overdose of sodium pentobarbital (40 mg/kg) and transcardially perfused with saline followed by 4% paraformaldehyde in phosphate buffered saline. The brain was extracted and further post-fixed for 24 h in 4% paraformaldehyde.

### Histological processing

Perfused brains were cryoprotected in increasing concentrations of glycerol (5–15% in 4% PFA), then sectioned on a cryostat at 50 μm thickness. Every tenth section was mounted unstained for fluorescence microscopy. These sections were dried and coverslipped with di-n-butyl phthalate xylene (DPX) following quick dehydration (2 × 100% ethanol) and immersion in xylene. Adjacent series of sections were stained for Nissl, myelin (Gallyas, 1979), and cytochrome oxidase (Wong-Riley, 1979).

### Microscopy and photography

Fluorescence labeled sections were examined unstained using a Zeiss Axioplan fluorescence microscope, and labeled cell bodies were plotted with an X-Y stage digitizer (MD-3, Accustage) and associated software (MD-Plot, v. 5.3). Photographs of selected tissue sections and injection sites were obtained using a Zeiss ICC5 camera. The resulting images were cropped, adjusted for level, brightness, and contrast, and re-sized using Adobe Photoshop.

### Data analysis

**Figure 1:**
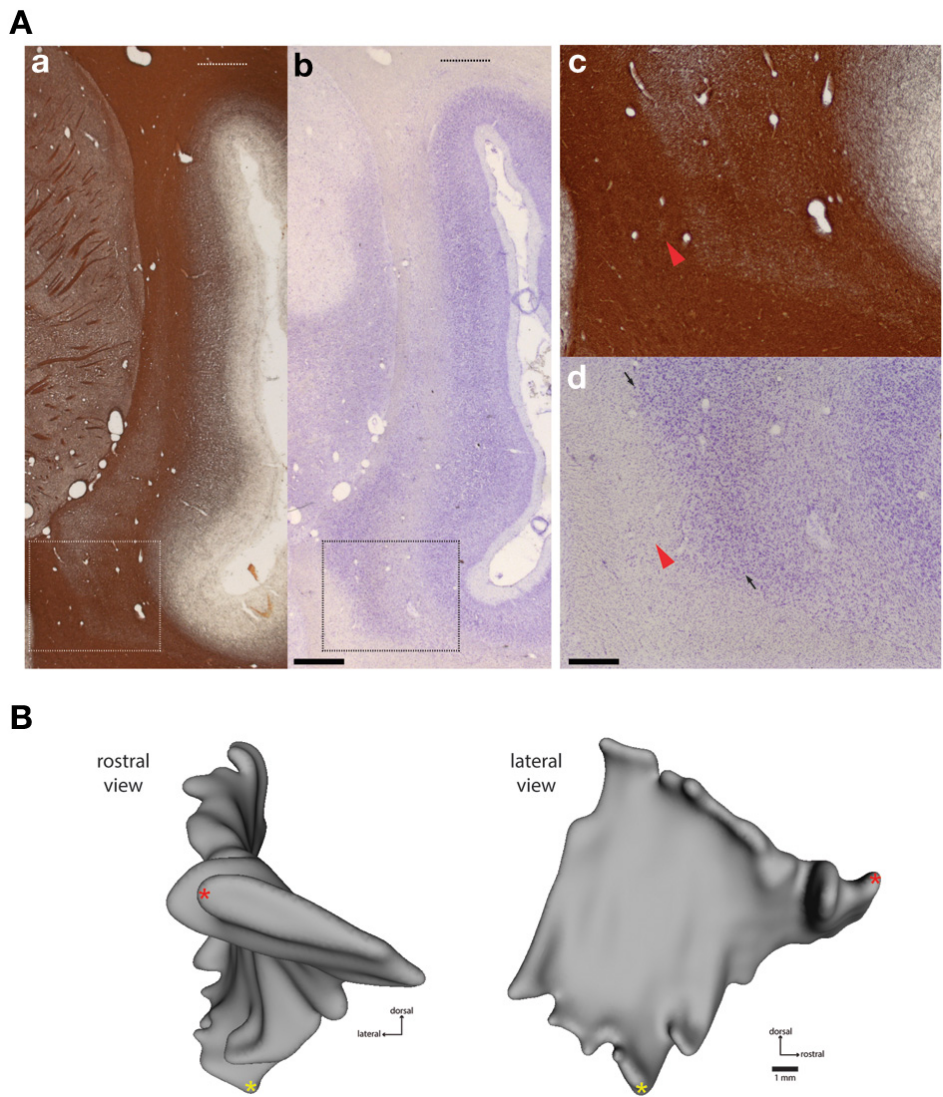
**Histological boundaries of *Cebus* claustrum**. **(A)** Myelin **(a,c)** and nissl **(b,d)** stained frontal sections showing the dorsal and ventral extent of the claustrum at approximately A-P +13.0 in the atlas of Eidelberg and Saldias (1960). Dashed lines in the low magnification images **(A)** show the maximum dorsal extent of the claustrum, which is difficult to appreciate in most histological preparations. The rectangular outlines in **(a,b)** indicate areas shown at high magnification in **(c,d)**. Panels **(c,d)** show the difficulty of accurate estimation of the ventromedial boundary of the claustrum in standard histological preparations. Red arrowheads show a small cluster of cells separated from the putative claustrum boundary by a portion of the external capsule white matter. Small arrows in **(d)** show the approximate boundary between the dorsal endopiriform nucleus and the insular claustrum as estimated from the histological characteristics of the marmoset (Paxinos et al., 2012). Detailed cytoarchitectonic information is not currently available for *Cebus*, so this parcellation should be considered provisional, pending identification of reliable markers of internal and external claustrum boundaries in this species. Scale bars = 1 mm in **(b)**; 0.2 mm in **(d)**. **(B)** shows frontal (left) and lateral (right) views of case FR01, modeled as described in Materials and Methods. The lateral view was used as the template for heat mapping of tracer distribution in subsequent figures. Red and yellow asterisks provide common points of reference for the rotated views.

Digital files containing cell count and position information were processed in Adobe Illustrator CS6, which was used to extract and align the claustrum outlines and surgical schematics. A three-dimensional model of the claustrum from case FR01 was created using manually aligned Nissl-stained sections with mid-thickness drawings, resulting in a series of contours that were then reconstructed into a 3D triangular mesh (Figure 1B), using the program CARET (Van Essen et al., 2001). The lateral view of this 3-dimensional model was then traced and smoothed in Illustrator, and overlaid with a 200 × 200 μm square grid, which was used as a template for plotting cells from each case, in order to facilitate comparison across injections. Each case was normalized to the maximum dorsal-ventral distance of the claustrum sections, and the grid was subsequently applied across all sections (24–26 sections per case). Cells within each grid square were counted and translated to a “heat map.” Color scales were derived by setting the low value to 20% of the respective color on the CMYK color scale, with the 100% value as the maximum (e.g., 20–100% yellow for the minimum-maximum range) for labeled cell density within each grid square. In practice, the cell density across all cases ranged from 1 cell to approximately 25 cells/grid, with the vast majority of grid squares containing fewer than 5 cells. Although this method yields a valuable display for comparison across injections and cases, it necessarily introduces some distortions, especially at the extreme dorsal and ventral portions of the map, where the *Cebus* claustrum varies the most in its medial-lateral extent. Moreover, the relative medial and lateral positions of labeled cells are lost in this flattened display. The distortion was considered acceptable in this study, as there were no cells in the dorsal-most or dorso-lateral portions of the insular claustrum in any of the cases studied. Spatial separation of cells in the ventral regions of the insular claustrum was observed, but this information is not captured in the flattened 2-D map format.

## Results

### General findings

The cytoarchitectonic and myeloarchitectonic characteristics of the *Cebus* monkey claustrum have not been previously described in detail. The appearance of the claustrum in frontal sections is generally consistent with that of other commonly used laboratory primate species, including the macaque (Pearson et al., 1982; Kowianski et al., 1999), vervet monkey (Kowianski et al., 1999), and marmoset (Burman et al., 2011a; Paxinos et al., 2012). One morphological difference between the *cebus* monkey and the marmoset is the dorsolateral extension of the insular claustrum into the white matter of the parietal operculum overlying the lateral sulcus, as shown in Figure 1. This is not observed in marmosets, but is present in macaques (Baizer, 2014) and humans, though the functional significance and cortical connectivity of this region remain poorly characterized.

Definition of claustrum borders with respect to the adjacent white matter tracts was clearest in myelin-stained sections (Figures 1Aa,c), with the claustrum appearing as a region of lightly myelinated tissue between the external and extreme capsules. The dorsolateral extension of the claustrum was evident in both Nissl and myelin stains, although it was faint (Figures 1Aa,b, upper dotted lines). In addition, precise determination of the rostral and ventral boundaries of the claustrum was difficult, especially at the rostral-caudal level, where it converges with the anterior insula, consistent with findings from both rodent and other primate species (Figure 1; Mathur et al., 2009; Paxinos et al., 2012). In several sections, small clusters of cells were located away from the apparent medial boundary of the claustrum (Figures 1Ac,d; red arrows). In caudal sections, the boundary between the dorsal endopiriform nucleus and insular claustrum was best appreciated in Nissl stained sections (dark arrowheads in Figures 1Ad). The general location of this boundary is consistent with the demarcation reported for the marmoset (Paxinos et al., 2012), but the presence of detached cell clusters (green arrowheads in Figures 1Ac,d) precluded volumetric measurement or direct comparisons between species. Cytochrome oxidase was not particularly useful for delineation of either boundaries or internal compartments of the claustrum (data not shown).

Tracer injections were deemed successful if the main tracer deposit was predominantly confined to an area of cortical gray matter which could be clearly localized by cytoarchitectonic and myeloarchitectonic characteristics, and for which long range transport of the tracer material could be definitively established by the presence of labeled cells in thalamic nuclei, cortical areas far removed from prefrontal cortex, or the homotopic contralateral cortical hemispheres. Nine successful tracer injections were placed in three monkeys. The majority of tracer deposits targeted the frontal pole (area 10). One deposit was placed in the rostral dorsolateral prefrontal cortex (area 9). Two injections were placed in area 12, one in the orbital subdivision (area 12o; case FR04-DY) near the border with the lateral subdivision of area 11, and one in ventrolateral prefrontal area 12 (FR02-DY). Double-labeled neurons were not observed, although it is still possible that these exist in small numbers.

As viewed from the lateral aspect, the *Cebus* claustrum is shaped like a distended rhomboid, slightly elongated on the rostral-caudal axis. Because of the undulating structure of the claustrum, this view has been used to demonstrate topography of cortical projections in previous studies (Pearson et al., 1982), and we employed it in this study to map the distribution of retrogradely labeled neurons, as detailed in the Materials and Methods.

### Frontal pole connections

**Figure 2:**
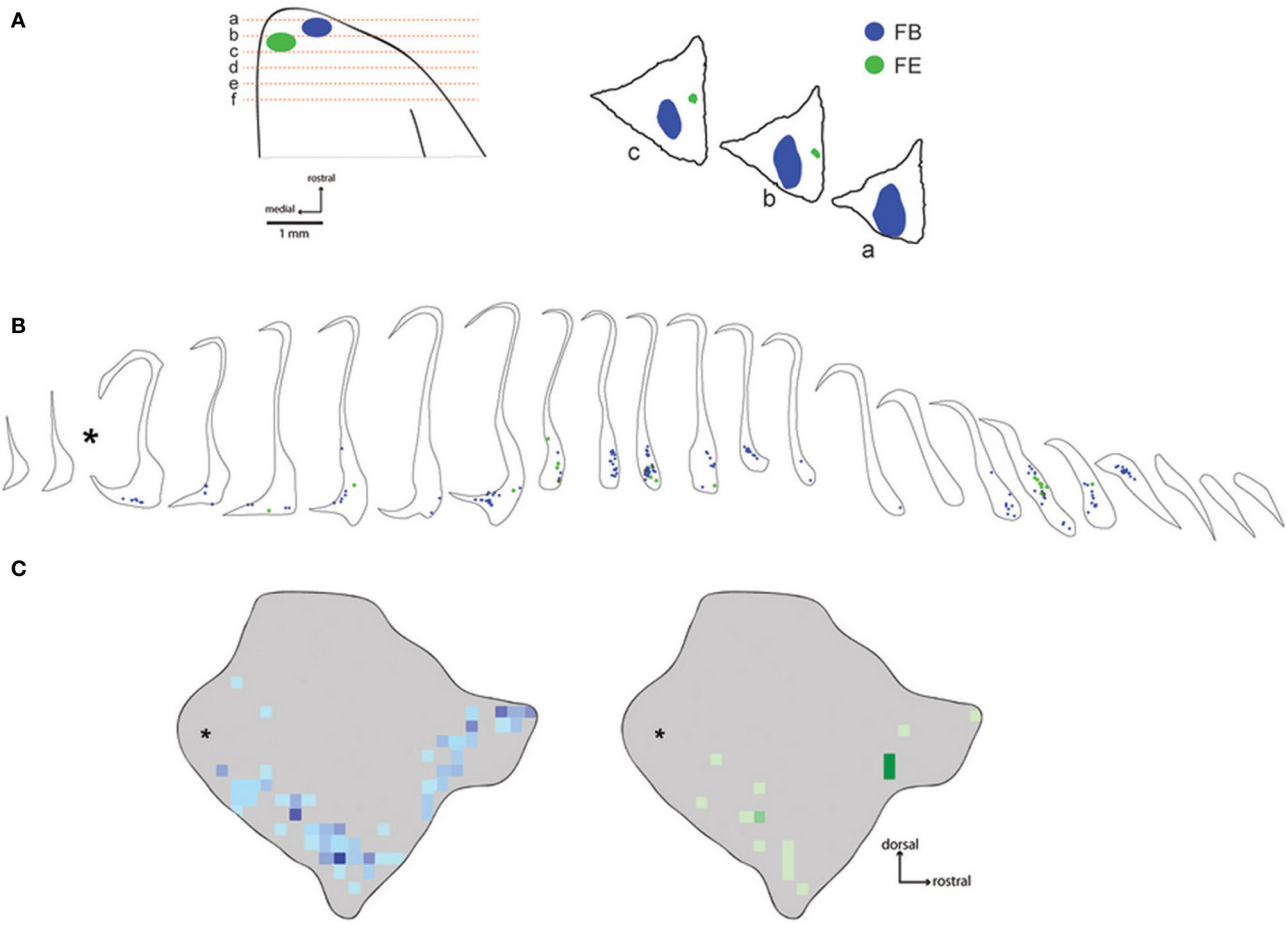
**Distribution of labeled neurons following injections in area 10**. The placements of fast blue and fluoroemerald injections into the frontal pole are shown in **(A)**. Coronal section locations are shown schematically on the left, while the positions of the main tracer deposits are shown in the digitized tracings on the right. Note that the fluid volume of the fluoro emerald (FE) injection in this case (FR02) was quite large (1 μl, Table 1), but the largest fraction of the deposit was located in the histologically stained sections between the unstained fluorescence sections plotted in this figure. Panel **(B)** shows the location of each of the labeled neurons from these injections in coronal sections of the claustrum. The corresponding heat maps are shown in **(C)**, illustrating the overall distribution of labeled cells overlaid on the lateral view of the 3-D model of the *Cebus* claustrum. Asterisks indicate the position of approximately 2 sections lost at the interface of the rostral and caudal tissue blocks during tissue cutting.

**Figure 3:**
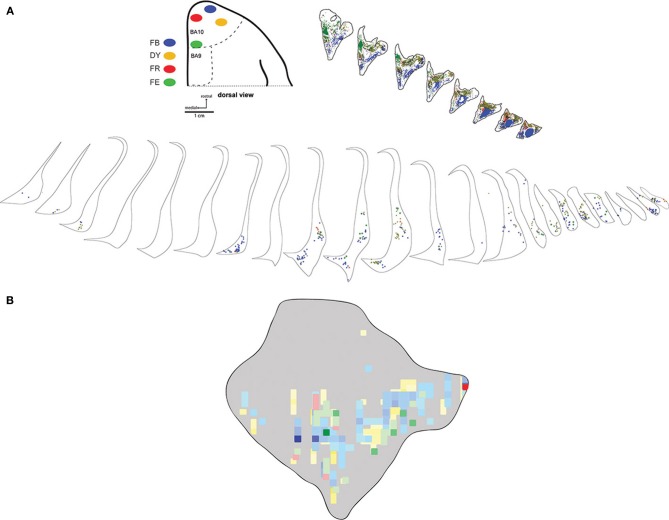
**Medial-lateral gradient of claustrum projections**. Locations of 4 tracer injections in area 10 of case FR01 are shown schematically in **(A)**. The distributions of labeled neurons for each tracer are overlaid in the heatmap format in panel **(B)**. Scale bars = 1 cm in **(A)**; 1 mm in **(B)**. FB, fast blue; DY, diamidino yellow; FR, fluoro ruby; FE, fluoro emerald.

Six injections were placed in the frontal pole region of two monkeys. The resulting distribution of labeled neurons in the ipsilateral claustrum is shown for a representative case (FR02) in Figure 2, which received two injections within area 10. Both injections resulted in patches of retrogradely labeled cells in the claustrum, which occupied a ventral position across multiple levels of the rostral-caudal axis. Areas of particularly dense clustering of labeled neurons were observed in the rostral and middle levels of the claustrum. However, no labeled cells were observed in the dorsal part the claustrum. The medial FE injection yielded far fewer labeled cells than the central FB injection, with the cells clustered into smaller areas (Figures 2B,C); these were completely encompassed within the area containing FB label. This pattern of increased claustrum label density following tracer injections in more rostral and lateral portions of area 10 was also evident in the second case, in which three of the injections were well contained within area 10 (FR01- FB, FR, DY), and one injection was located near the boundary with area 9 (FR01-FE). The distributions of tracer resulting from those injections are summarized in the heat map in Figure 3. The central and lateral injections (FB and DY, respectively) labeled a much broader area of ventral and medial claustrum than the medial injections (FE and FR), both in terms of overall tissue area and density of labeled neurons. Both medial injections yielded only isolated labeled cells in the claustrum. Whether this trend reflects functional differences within area 10, or different transport properties of the dextran-based tracers (FR and FE) will require further study. However, both of the medial area 10 injections resulted in long-range transport of tracer, confirmed by the presence of labeled neurons in various thalamic nuclei (data not shown).

### Dorsal prefrontal connections

**Figure 4:**
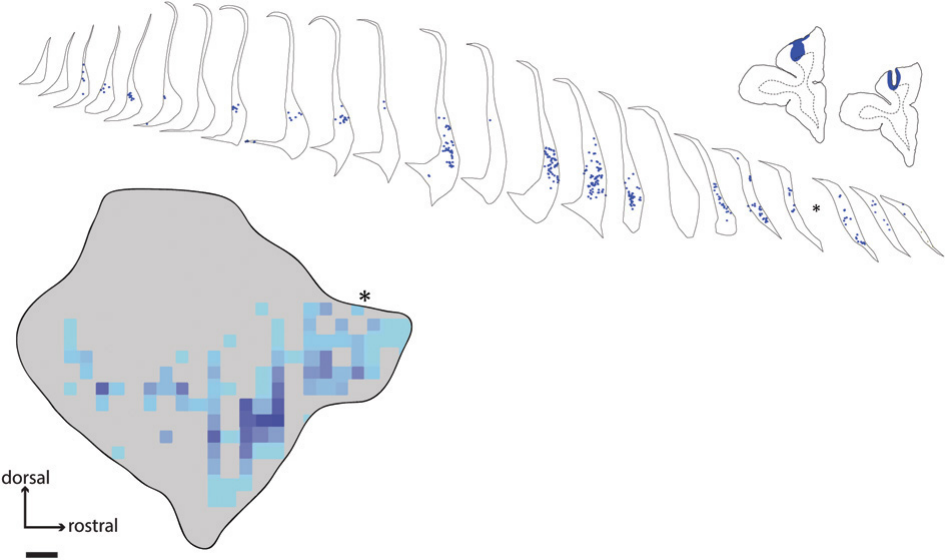
**Distribution of labeled neurons in claustrum following tracer injection in area 9**. Line drawings show placement of fast blue injection in case FR04, along with the resulting retrogradely labeled cell bodies in the ipsilateral claustrum. Asterisk indicates the position of a single tissue section lost due to slide damage during coverslipping. Scale bar = 1 mm.

The dorsal prefrontal region includes areas 8, 9, and 46 (Petrides and Pandya, 1999; Sallet et al., 2013). In this study, a large fast blue injection was deposited in area 9 of one animal (case FR04-FB), which yielded patches of retrogradely labeled neurons in a band which closely tracked the distribution of label observed following injections into the frontal pole, although in a slightly more dorsal position within the claustrum. A discrete, longitudinal patch centered in the rostral part of the claustrum was the dominant pattern, with isolated cells and scattered small patches extending along the ventral border to the caudal terminus (Figure 4). Consistent with the pattern observed following injections in area 10, the dorsal and dorsolateral parts of the claustrum were devoid of label, and no interhemispheric projections from the contralateral claustrum were evident. A single isolated cell body was observed in the mid-dorsal region of the contralateral claustrum following this area 9 injection.

### Ventral lateral prefrontal and lateral orbitofrontal connections

**Figure 5:**
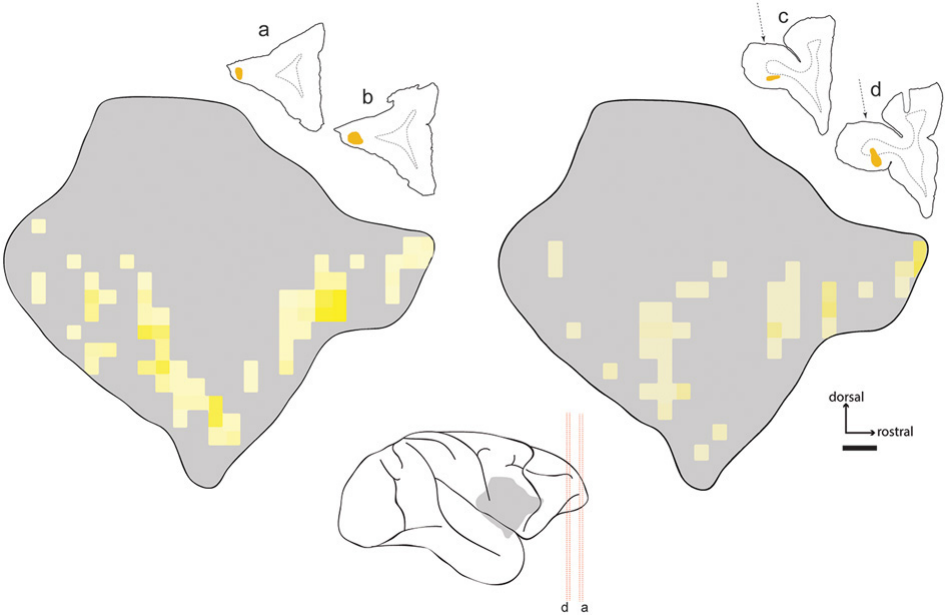
**Distribution of labeled neurons in claustrum following injections in area 12**. Heat map projections for two injections of diamidino yellow (DY) in area 12 (cases FR02- left; FR04- right). Line drawings show injection locations in frontal sections. Center schematic image shows location of claustrum relative to tissue sections and major sulci of the *Cebus* brain (gray silhouette). Red dashed lines correspond to tissue sections as marked. The dotted arrows on the right indicate the approach used for injection of area 12o, which traversed the cortical white matter overlying that area. Implications of this approach for interpretation of the data are described in the text.

Two injections were placed in subdivisions of area 12. One DY injection was deposited in the ventral lateral prefrontal cortex (rostrolateral area 12; case FR02-DY), while the other was predominantly in orbital area 12 (case FR04-DY). In the latter case, the injection site obscured the likely cytoarchitectural boundary with area 11, so we cannot definitively exclude the possibility that some of the tracer was deposited in this area. However, the distribution of labeled neurons from both injections was qualitatively similar, as shown in Figure 5. As observed following prefrontal injections in areas 9 and 10, the majority of labeled cells were observed in a band running along the ventral part of the claustrum, with no cells in the dorsal or dorsolateral insular claustrum.

### Summary of connections

**Figure 6:**
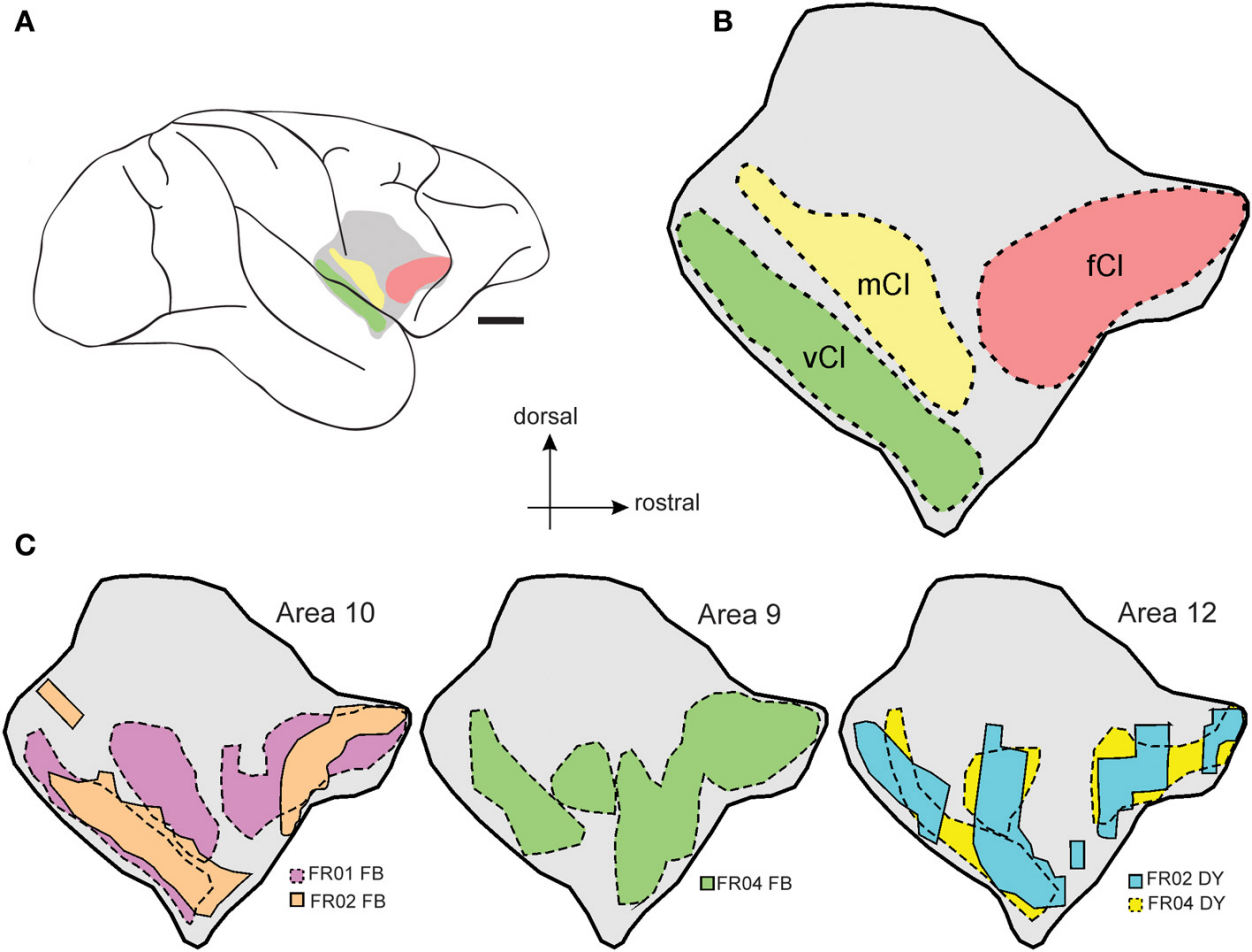
**Summary of claustrum-prefrontal projections**. Regions of the *Cebus* claustrum as defined by Gattass et al. (this volume) are schematically displayed as color coded regions in **(A,B)**. **(C)** illustrates the smoothed outline of the maximum extent of each patch of labeled cells within the claustrum for each of the prefrontal cortex injections included in this study. Scale bar = 4 mm in **(A)**.

The full extent of claustrum projections to prefrontal cortex is summarized in Figures 6A–C, which shows the relative position of the claustrum in lateral view (Figure 6A), as well as some of its proposed subdivisions (frontal- fCl, middle- mCl, and ventral- vCl; see Gattass et al.; this volume) within which prefrontal connections originated (Figure 6B). A smoothed representation of the extent of labeled cells originating from each case is shown in Figure 6C, which indicates the degree of homogeneity observed from injections into specific prefrontal areas.

## Discussion

The claustrum in most mammals has been broadly divided into a dorsal compartment, the “insular claustrum” or simply “claustrum,” and the endopiriform nucleus, which is in turn divided into dorsal and ventral components (Paxinos et al., 2012). In other primate species, as well as in cats, the majority of connections from sensory and association cortex are confined to the insular region of the claustrum, while the endopiriform nucleus is largely connected with olfactory and entorhinal cortex, along with limbic subcortical nuclei. Our data in the *Cebus* conform to this pattern, with the overwhelming majority of projections to frontal pole and dorsolateral prefrontal areas arising from insular claustrum.

### Organization of claustrum-prefrontal connections

The distribution of retrograde label in the claustrum following prefrontal injections in *Cebus* was largely consistent with our previous findings in the marmoset (Burman et al., 2011a), and with the topography of claustrum-prefrontal projections reported in macaques (Pearson et al., 1982). In particular, labeled neurons following injections in areas 9 and 10 were concentrated in a band located along the ventral portion of the claustrum. While the injection in area 9 labeled cells primarily in the rostral part of the claustrum, those in area 10 resulted in strong label in a second, more caudal cluster (compare Figures 2, 4). Claustrum projections to area 12 originated from a similar territory. Labeled neurons were largely absent from the narrow dorsal region of the claustrum, medial to insular cortex, and were completely absent from the extreme dorsal and dorsolateral regions, which have been reported to contain the bulk of claustral neurons projecting to somatomotor cortex in the macaque (Pearson et al., 1982; Minciacchi et al., 1991). It should be noted that the injection of area 12o (Case FR04-DY) likely involved the white matter dorsal to this area, as the injection needle passed through the overlying tissue. However, we are confident that this did not affect the validity of our observations to an appreciable extent, given the lack of non-specific label in both cerebral hemispheres, which is generally observed in cases of significant white matter intrusion, and the similarity of the pattern of claustrum label resulting from this injection to the other area 12 case (case FR02-DY), in which the white matter was not involved.

With the exception of an isolated FB neuron from an injection in area 9 (case FR04-FB), retrogradely labeled neurons were restricted to the ipsilateral claustrum. This is inconsistent with the pattern of interhemispheric connections observed in the rat, e.g., between claustrum and the frontal eye field (Smith and Alloway, 2014), and other prefrontal areas (Sloniewski et al., 1986; Smith and Alloway, 2010). This difference is unlikely to be related to the directionality of the tracers employed, as both FB and DY were employed by Slownieski et al., and resulted in bilateral labeling. In primates, interhemispheric claustral connections have been reported in the mouse lemur (Park et al., 2012), using high resolution diffusion tensor imaging, and in humans, using constrained spherical deconvolution tractography (Milardi et al., 2013). However, the nature of these methods does not allow disambiguation between projections to or from the cortex (see also Kunzle, 1975). Our data suggest that the interhemispheric claustrum-prefrontal projections in primates are qualitatively weaker than those of the rodent.

Consistent with results in the marmoset (Burman et al., 2011a), tracer injections into medial area 10 of *Cebus* yielded far fewer labeled cell bodies in the claustrum than injections in the dorsal and lateral portions of area 10. Whereas that result is to be considered preliminary based on the small number of injections and potential variation in transport of different tracer types, these results are in line with the hypothesis that differences in connectivity with the claustrum are related to other functional differences described between the lateral and medial subregions of area 10 (Ongur et al., 2003; Burman et al., 2011a,b). Area 10 is a cortical region that has expanded significantly across primate species, including humans (Semendeferi et al., 2001), and interestingly, the lateral portion of area 10 undergoes considerably greater postnatal expansion in humans than does the medial portion (Hill et al., 2010). A systematic review of the area 10 literature by Gilbert et al. (2006) found evidence for a significant difference in activation of medial vs. lateral area 10, with the lateral region exhibiting greater responses to tasks involved in episodic retrieval of non-emotional content. Whether the apparent difference in claustrum connectivity with the lateral vs. medial area 10 is present in humans and potentially correlated with the functionality of this area is an open question, which may be addressable using high definition tractography or similar approaches. Somewhat curiously, no double labeled neurons were observed in the claustrum in FR04 or any of the other cases examined, suggesting that projections from individual claustrum neurons to prefrontal areas in *Cebus* are restricted to relatively small cortical populations. The apparent absence of double labeled cells is especially surprising in case FR04, in which the tracers were placed in close proximity within a relatively small cortical area. It will be interesting to see if this pattern holds among tracer injections in widely disparate cortical areas, e.g., simultaneous tracer placement in PFC and cingulate or parietal areas, for instance. In the macaque, Selemon and Goldman-Rakic (1988) reported overlapping fields of terminal label from simultaneous parietal and prefrontal injections using anterograde tracer injections.

The distribution of labeled neurons in claustrum following the area 9 injection was largely consistent with previous studies of the macaque (Pearson et al., 1982; Saleem et al., 2014) and marmoset. Damage or hypoactivation of this area is often reported in association with schizophrenia and impaired working memory (Barch et al., 2003). The dense projection we observed from the rostral and ventral claustrum to area 9 suggests a possible focus for future investigations of claustrum involvement in prefrontal function in both normal and pathological function, and the similar topography of this projection across primate species suggests that widely used laboratory primate species could be effective models of both of these states.

Activity in ventrolateral prefrontal cortex, which includes area 12 (according to Carmichael and Price, 1996; note that this region overlaps with area 47 in other nomenclatures Petrides and Pandya, 2002; Paxinos et al., 2012), is also associated with retrieval of information from memory, but not with maintenance or monitoring of that information post-retrieval, which is associated with areas 9 and 9/46 (Cadoret et al., 2001). Thus it is likely that the functional interaction between the claustrum and prefrontal cortex overlaps both working memory- and retrieval- dependent processes.

### Relationship of claustrum-prefrontal projections to claustrum connections with sensory and association areas

Previous tracing studies of claustral projections in primates have shown localized sensory cortical connections within the body of the claustrum (Reviewed in Druga, 2014; visual cortex- Tigges et al., 1982; Doty, 1983; Baizer et al., 1997; Gattass et al., 2014; somatosensory cortex- Pearson et al., 1982; Minciacchi et al., 1991; auditory cortex- Pearson et al., 1982; Smiley et al., 2007; Reser et al., 2009). As mentioned above, it is currently impossible to draw complete homologies of the claustrum anatomy of primate species, though some general patterns have emerged. Projections to visual areas are largely confined to the caudal and mid-dorsal regions of the claustrum. In contrast, motor and somatosensory projections are heavily concentrated in the dorsal and dorsolateral portions of the claustrum, with virtually no overlap between the areas of somato-motor connectivity (Pearson et al., 1982; Minciacchi et al., 1991) and the distribution of prefrontal label we observed. Auditory connections are somewhat more difficult to characterize. We have observed sparse auditory cortex connections in the marmoset (Reser et al., 2009), and other groups have reported claustrum projections to auditory areas in the macaque (Smiley et al., 2007). Where topographic information has been provided, auditory connections were restricted to the ventral claustrum (Pearson et al., 1982). Thus, it appears that there is relatively little overlap among the major sensory cortical areas and PFC projections. This roughly concords with the description of claustrum connections in the human as reported by Fernandez-Miranda et al. (2008), allowing for differences in claustrum topology across species. Pearson et al. (1982) reported that labeled cells from injections in area 22 (non-primary auditory cortex) in the macaque were concentrated along the ventral portion of the middle and caudal claustrum. Based on the demarcation of the injection zone in that report, it is likely that the target tissue included part of the temporal lobe polymodal association cortex. The precise relationship of claustrum projections to association areas and respectively, auditory and visual sensory areas along the primate temporal lobe, remains to be determined.

### Hypothesis: possible claustrum involvement in switching between resting state networks

As discussed below, there are intriguing parallels between the observed anatomical connectivity of the claustrum and the prefrontal components of several of the known cortical resting state networks. Here we introduce the hypothesis that one function of the primate claustrum may involve mediation or modulation of resting state network activity.

The identification of synchronously oscillating patterns of regional blood flow and synaptic activity across cortico-cortical and subcortical-cortical networks in recent years has forced a re-examination of what occurs in the brain during periods of presumed inactivity. Approximately a dozen resting state cortical networks (Mantini et al., 2011; Van Den Heuvel and Sporns, 2013) have been identified in primates, including: the default mode network (DMN; Greicius et al., 2003; reviewed in Buckner et al., 2008); the central executive network (Damoiseaux et al., 2008); the fronto-parietal control network (Dosenbach et al., 2007; Vincent et al., 2008); and the salience network (Downar et al., 2002; Seeley et al., 2007).

Our data show that prefrontal areas which are part of the salience network (area 12) and areas which are not (9, 10) receive input from the same region of the claustrum (the rostral and ventral region), though the precise topography of inputs requires more detailed investigation. Specifically, it will be necessary in future studies to determine if individual claustrum cells project to multiple areas of prefrontal or other cortex, i.e., whether they could provide input to multiple cortical functional networks. The overlap in topography of input from the claustrum to these areas suggests to us that the circuitry of the claustrum-prefrontal connection would ideally position the claustrum as a modulator or “switch” that could desynchronize or terminate correlated activation of DMN-related areas when external cues require activation of the various task-positive networks. Indeed, the time-series analysis of Seeley et al. (2007) showed that the component cortical areas of the salience network exhibited weak correlation over time, suggesting that the temporal structure of network activity could be dictated by one or a very small number of hub areas. The claustrum is ideally positioned, in terms of connectivity and anatomy, to act in this capacity. Additional anatomical data which would be required to assess this hypothesis includes mapping of claustrum connections with other known network hubs, which include (for the DMN) ventromedial prefrontal (Damoiseaux et al., 2008) and subgenual anterior cingulate cortex (Mantini et al., 2011), the precuneus, and especially posterior cingulate cortex (Damoiseaux et al., 2008; Belcher et al., 2013). Extensive study will be required to assess the hypothesized involvement of the claustrum in DMN and/or other cortical networks, and to determine whether the claustrum acts in isolation or in concert with other cortical or subcortical structures.

### Possible limitations of this study

There are several aspects of this study which must be considered in evaluation of our results and conclusions. First, the homology between brain areas of different primate species is difficult to establish, especially as the size and function of the various regions is known to have changed across primate evolution (Semendeferi et al., 2001; Chaplin et al., 2013), with a general trend toward segregation between areas and networks with increasing cortical size (Changizi and Shimojo, 2005). In both body mass and brain volume, the *Cebus* monkey is at least 10 times larger than the species we have studied most recently, the marmoset, so it is reasonable to assume that some functional changes across prefrontal areas have occurred. A second key consideration is our poor understanding of the functional architecture of the claustrum itself, especially in primates. Although recent advances have been made in identification of anatomical and neurochemical markers of the claustrum (Arimatsu et al., 2009; Mathur et al., 2009), this approach has not yet yielded reliable results in primates. Thus, it remains difficult to infer claustrum functions from compartmentalization or topographic organization of cortical connections. Finally, testing hypotheses regarding claustrum involvement in cortical functional networks will require a more precise survey of which cortical areas are components of specific networks, and reconciliation of the anatomical definitions of those areas with areas of increased or decreased activity in functional studies, which will likely require intensive future study and revisitation of existing datasets.

### Conflict of interest statement

The authors declare that the research was conducted in the absence of any commercial or financial relationships that could be construed as a potential conflict of interest.
